# Facial transplantation: A concise update

**DOI:** 10.4317/medoral.18552

**Published:** 2012-12-10

**Authors:** Pedro Infante-Cossio, Fernando Barrera-Pulido, Tomas Gomez-Cia, Domingo Sicilia-Castro, Alberto Garcia-Perla-Garcia, Purificacion Gacto-Sanchez, Jose-Maria Hernandez-Guisado, Araceli Lagares-Borrego, Rocio Narros-Gimenez, Juan D. Gonzalez-Padilla

**Affiliations:** 1 Department of Oral and Maxillofacial Surgery, Virgen del Rocio University Hospital, Seville, Spain; 2 Department of Plastic and Reconstructive Surgery, Virgen del Rocio University Hospital, Seville, Spain; 3 ….

## Abstract

Objectives: Update on clinical results obtained by the first worldwide facial transplantation teams as well as review of the literature concerning the main surgical, immunological, ethical, and follow-up aspects described on facial transplanted patients. Study design: MEDLINE search of articles published on “face transplantation” until March 2012. Results: Eighteen clinical cases were studied. The mean patient age was 37.5 years, with a higher prevalence of men. Main surgical indication was gunshot injuries (6 patients). All patients had previously undergone multiple conventional surgical reconstructive procedures which had failed. Altogether 8 transplant teams belonging to 4 countries participated. Thirteen partial face transplantations and 5 full face transplantations have been performed. Allografts are varied according to face anatomical components and the amount of skin, muscle, bone, and other tissues included, though all were grafted successfully and remained viable without significant postoperative surgical complications. The patient with the longest follow-up was 5 years. Two patients died 2 and 27 months after transplantation. Conclusions: Clinical experience has demonstrated the feasibility of facial transplantation as a valuable reconstructive option, but it still remains considered as an experimental procedure with unresolved issues to settle down. Results show that from a clinical, technical, and immunological standpoint, facial transplantation has achieved functional, aesthetic, and social rehabilitation in severely facial disfigured patients.

** Key words:**Face transplantation, composite tissue transplantation, face allograft, facial reconstruction, outcomes and complications of face transplantation.

## Introduction

Since the first face transplantation (FT) was successfully completed in November 2005 in Amiens (France) (1), the global clinical experience gained in France, China, USA, and Spain has shown that transplantation of facial structures is a feasible procedure in reconstructive surgery leading to general acceptance in the scientific world and society. FT is included in the composite tissue allotransplantation (CTA) concept that combines skin, bone, muscles, tendons, and nerves. To date, tissue transplantations have been described in hands, abdominal wall, tongue, larynx, face, esophagus, and knee (2). Although currently facial CTA is still in an experimental stage, the functional and aesthetic results that have been reported are very encouraging. Patients have achieved, to a great extent, significant improvement in appearance and largely recovered motor and sensory facial functions (3). By doing so, they have not only recovered essential functions such as breathing, talking, eating and oral competence, but also personal identity as well as social and family successful interaction.

TF has become an innovative surgical option indicated in patients in whom conventional reconstruction techniques have failed (4). In general terms, it has been used to solve clinical situations accompanied by severe bone and soft tissue facial loss resulting in serious aesthetic, functional and sensory deficiencies as a consequence of animal attacks, burns, gunshot injuries, neurofibromatosis type I, and cancer ablations, among others (5,6).

Although FT has progressed enormously in the last 6 years, the scientific community should be cautious given that clinical follow up involves little more than ten extremely selected cases. There are still many experimental research perspectives and clinical application in patients in order to define the scope and the true clinical effective therapeutic dimension of FT. In this paper, we review the results described by the first FT teams in the world, and analyze the most important clinical results obtained as well as the main surgical, immunological, ethical, and monitoring aspects that have been raised to date.

## Material and Methods 

We performed a bibliographic search until March 2012 in PubMed/Medline (http://www.ncbi.Nlm.nih.gov /PubMed) and then conducted a comparative analysis of published data on the first face transplantations. Inclusion criteria were as follows: English-language articles generated from the Medline database containing at least one case report of FT regarding clinical, diagnostic or therapeutic criteria. The initial search identified 30 articles that met these criteria (1,7-36). Some patient data have been repeatedly reported in more than one publication by several peer-review journals.

## Results

During the study period, clinical data of 18 patients with FT were reported. Table 1 and  Table 1 (continued) summarizes the main characteristics of the patients. Amongst the 18 patients included in the present study, a higher prevalence of men was found (15 cases). The mean age was 37.5 years, with an average age range between 27 and 59 years. Main etiological indications were gunshot injuries (6 patients), burns (5 patients), injuries caused by animal attacks (3 patients), neurofibromatosis type I (3 patients) and cancer ablation consequences (1 patient). All transplanted patients had previously undergone several conventional surgical reconstructive procedures which had failed (8,33,37). Altogether 8 transplant teams from 4 countries participated.

**Table 1:**
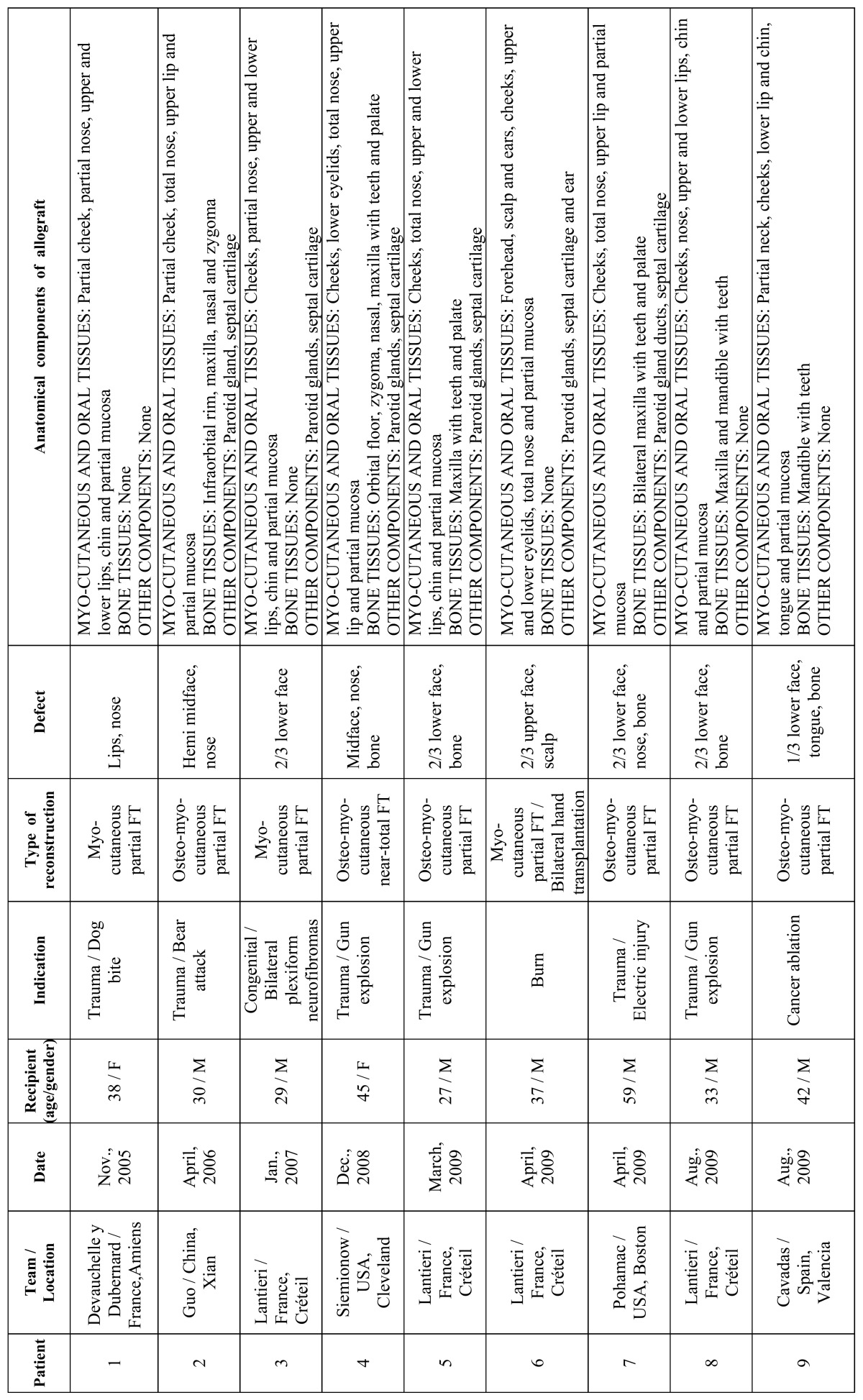
Summary of main characteristics of the FT performed between 2005 and 2011.

**Table 1 (continued):**
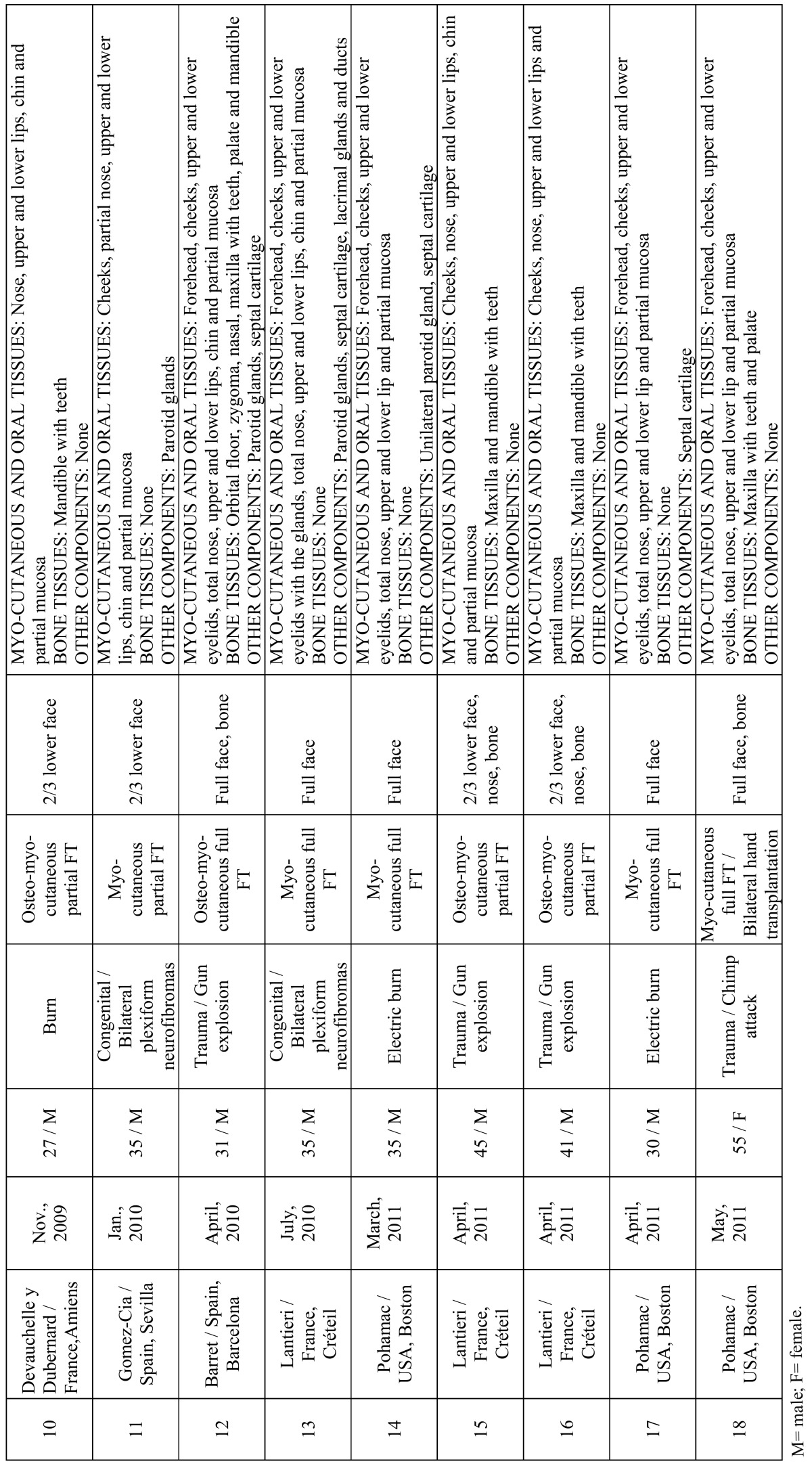
Summary of main characteristics of the FT performed between 2005 and 2011.

Reconstruction options were divided into 2 major categories based on the defect type: partial FT in 13 cases (including at least one third of the face) and full FT in 5 cases. Allografts were varied according to the anatomical components and the amount of skin, muscle, bone, and other facial tissues used ( Table 1,  Table 1 (continued)), but all were grafted successfully and remained viable after re-vascularization without significant postoperative surgical complications. The patient with the longest follow up was 5 years (35). Two patients died. The first one underwent FT in China during 2006 and died 27 months after transplantation (15). The other one was the first patient to receive a simultaneous face and bilateral hands transplant in France during 2009 and died 2 months after transplantation due to septic shock (19).

## Discussion

- Preclinical models of face transplantation

**Figure 1:**
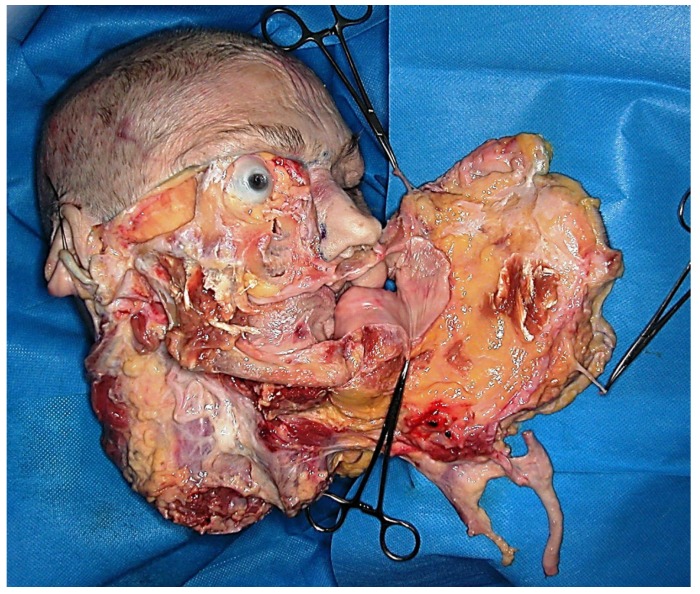
Allograft anatomical dissection involving the lower two thirds of the face.

Different worldwide transplantation teams have conducted research on facial CTA in animal models. The most frequently used were rats, although other models such as pigs and primates have been used (38). As in any other scientific innovation performed in the surgical field, pre-clinical studies developed on cadaveric models have been critical in order to find out the best way to remove soft tissues, facial bones, muscles, nerves and vessels from the donor with the minimal tissue ischemia (Fig. 1). Studies on cadavers have also been performed to mock the harvesting of segmental facial CTA including lower, middle, and/or upper parts of the face (12,19,29).

- Surgical considerations

Microsurgical procedures in FT are similar to those performed in other complex facial reconstructive surgical procedures (3). The key issue here focuses on the exact planning and execution of FT, always keeping in mind the adequate perfusion of the allograft, for which facial tissues vascularization and angiosomes knowledge are essential. All allografts were harvested in a single mono-block segment containing the CTA components of the donor (i.e.: facial osteo-myo-cutaneous tissues with mimic muscles, vessels, and motor and sensory nerves). Therefore, this ensures complete allograft re-vascularization by preserving muscle-cutaneous perforating vessels localized between the mimic muscles and the cutaneous component.

Facial allograft vascularization restoration may be achieved with relatively few vascular anastomoses. Most anastomoses were performed in large diameter vessels in order to minimize thrombosis risk (external carotids, jugular veins and thyro-linguo-facial trunks). Moreover, it has been shown that complete facial re-vascularization is possible to achieve by means of a single pedicle anastomosis of the facial vessels (22), as well as the viable re-vascularization of the maxilla, palate and mandible (3,13,32). Alternatively, our team conducted a temporary vascular anastomosis on the femoral vessels to achieve allograft heterotopic perfusion while performing the rest of the surgical procedures (33).

Some teams accessed the facial nerve via parotidectomy, dissecting the facial nerve trunk at the stylomastoid foramen output, making the nerve connection at the main trunk of the recipient and including the parotid glands in the allograft (19,33). Other teams made the nerve anastomosis in the facial nerve peripheral branches, performing an intraparotid nerve dissection (16,31). In partial FT, mental and infraorbital nerves were connected, while in full face transplantations connection of the supraorbital nerve was also performed (33).

In both full FT as well as transplantations involving the lower two thirds of the face, the nose and various segments of the facial bones may be included, performing the necessary osteotomies according to the recipient needs. Patients suffering from gunshot trauma usually require maxillary and/or mandibular reconstruction by means of a Le Fort II osteotomy combined with the mandible anterior segment removal, usually containing teeth (16,22,29,31). In these cases, subsequent osteosynthesis must be performed and dental occlusion ensured. The chin segment was also transplanted as a mono-block (26). Some teams found it difficult to achieve an adequate bone fixation due to anatomical differences between donor and recipient (6). In the near future and based on CT images, simulation tools to perform preoperative procedures are developing to virtually guide the osteotomy, facilitate maxillo-mandibular insertion and guide dental occlusion.

Some teams have reported a significant blood loss during the procedure. The second patient allograft performed in China resulted in 5 liters blood loss (10). Lantieri et al. (11) described transfusion requirements of 10 or more units of packed red blood cells in each of their first four patients. There was also a severe blood loss in our patient case (26), as 24 packed red blood cells units were transfused resulting in a post-transplant dilutional coagulopathy. It is interesting to note that our patient developed rhabdomyolysis after transplantation, which was attributed to both massive blood loss and to an intra-operative immobilization long period (18).

- Preparation of donor and recipient before transplantation

**Figure 2:**
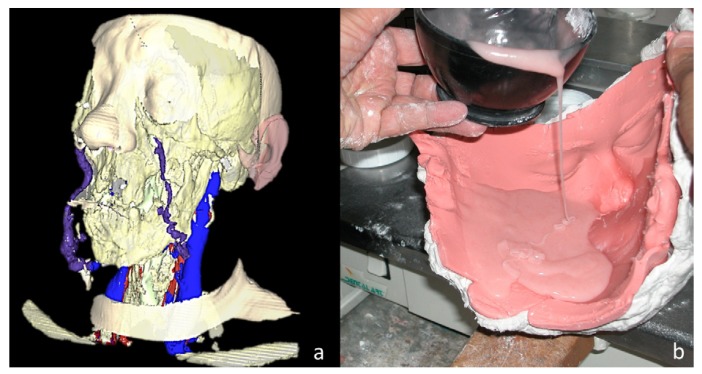
FT of the lower two thirds of the face. (A) Recipient preoperative planning using a three-dimensional reconstruction performed with the AYRA software (formerly VirSSPA, Andalusian Health Service, Seville, Spain). (B) Preparation of the resin mask after obtaining an alginate donor mold.

Donors and recipients were matched for race, sex, blood type, HLA and skin colour (3-6,37,39). Anthropometric measurements of head size were used as a reference. Measurements based on virtual reality technology have also been used by some teams (Fig. 2) (33). In all cases, a brain death donor and family consent were needed.

A thorough psychological evaluation of the recipient is essential before considering to be included as a FT program candidate (6,32). Clear contraindications are affective, behavioral, cognitive and/or perception disorders that impair the patient’s ability to follow lifelong immunosuppressive therapy. Informed consent prior to partial or full FT requires a clear understanding not only regarding surgical risks, but also immunosuppressive therapy risks and the likely possibility of CTA rejection.

- Recovery procedure from the donor 

In the majority of cases, the standard systematic recovery of allografts was from brain-death heart-beating donors (1,16). In the case of multi-organ procurements, the majority of worldwide FT teams harvested the “face in the first place.” Bueno et al. (24) demonstrated the feasibility of harvesting the facial allograft at the same time as solid organs in close collaboration with other transplant teams, performing approximately half of the dissection of the facial allograft before the removal of the solid organs and the rest shortly after the cardiac death. Our team, however, performed the entire allograft recovery after donor’s cardiac death, beginning shortly after thoracic and abdominal organs removal (25). In cases where allograft recovery and insertion was performed in different hospitals, transport has to be done safely in the organ preservation solution inside a refrigerator with ice and as quickly as possible to limit tissue ischemia time (32,36).

A crucial aspect to consider in FT is the donor’s face restoration once the recovery is completed. In solid organ transplantation, organs removal from the abdominal cavity or chest does not cause a visible donor deformity. However, a facial CTA removal involves a very distorting defect in the donor’s face. In the whole Europe, Transplant Coordination Authorities undertake to restore donor’s body to make it look as normal as possible before returning to the family (29,33). The donor mask making process (maxillofacial epithesis) starts by making an alginate mold of the patient’s face (Fig. 2). Transplant teams have restored donor faces with resin or silicone masks (36). In most cases, a make-up resin mask has been manufactured in the laboratory simultaneously while the recovery of organs and tissues (33).

- Transplant indications

The most common FT indication was to restore the facial lower two thirds structures, especially the perioral area (which affects lips, cheeks and chin), including in some cases the forehead, eyelids and scalp, as well as the maxilla, mandible and teeth (39). The most frequent disfigurement causes were burns, gunshot trauma, animal bites, trigeminal nerve plexiform neurofibromas in the neurofibromatosis type I context, and tumor ablation sequelae (3). Lantieri (6) suggested that FT indications should not be based solely on the defect etiology, but it also should consider three other aspects: the anatomical deficit to be reconstructed, patient characteristics (such as quality of life, health, immune-sensibilization, psychosocial support, etc.), and the transplant team experience.

- Immunology

Skin is the largest component of FT, and it is well known to have high immunogenic property. For that reason, after a FT, it is inevitable that episodes of acute and chronic rejection occur (2,7). Nowadays and, in order to prevent FT rejection, non-specific induction of overall immunosuppression is used as a triple therapy based on postoperative administration of mycophenolate mofetil, tacrolimus and steroids. This immunosuppressive regimen should be continued throughout life, which leads to toxicity risks, infectious complications (opportunistic infections with cytomegalovirus, herpes, etc.), metabolic complications (diabetes), nephrotoxicity, hypertension and tumors (37). Although a risk of chronic rejection theoretically exists, it is not known to what extent immunosuppressive treatment can cause CTA dysfunction over time and shorten life. Moreover, no case with clinical or histological evidence of chronic rejection has been reported (35).

To prevent episodes of FT rejection a systematic clinical monitoring is required regarding skin and/or oral mucosa biopsies (7). All FT recipients have experienced different acute rejection levels at some point (1,20,31). However, all rejection episodes were successfully resolved by increasing systemic immunosuppression (33).

Current research is focused on getting new immunosuppressive molecules that allow readjustment of drugs (such as steroids) and avoid the problems associated with chronic rejection. The investigations are being conducted mainly on the anti T cell antibodies, the development of more selective molecules with less toxicity to organs (kidney, liver) and the creation of a hematopoietic chimerism state. Furthermore, new immunosuppressants associations are being studied to reduce their doses; thus, being able to decrease toxicity. Some centers have used bone marrow infusion, thymoglobulin, anti-IL-2 receptor antibodies, and X-ray irradiation. Since FT skin is the most antigenic allograft part, topical treatment with tacrolimus and phototherapy have also been used (3).

- Functional and aesthetic results

FT aims to restore the speaking, swallowing and mimic musculature mobility functions as well as to provide aesthetic improvements allowing patients to conduct a normal social life. Although a systematic analysis of all cases cannot be performed due to each patient unique characteristic, results of the first FT as a whole are very convincing.

Unlike solid organ transplants, in which a metabolic function is found soon after re-vascularization of the organ, CTA is initially viable after reperfusion in the operating room, but its motor activity and sensitivity remain absent at the beginning. Therefore, in the first months of follow-up the challenge is nerve regeneration and muscle rehabilitation as well as the patient’s ability to reintegrate the allograft in the sensory and motor cortex at the central nervous system. Between 6-9 months after transplantation, patients returned to normal ranges in terms of warmth and cold, and recovered the discriminative facial sensibility. Active and passive lip movements were obtained between 6 and 12 months, but results differ between full and partial FT (8,16,31,37). Patients had been able to breathe through the nose, smell, chew, swallow, eat, and recover phonatory function and speech intelligibility. Recovery of facial movements allows them to express their feelings and gives these patients a certain facial expression. Aesthetic outcomes are satisfactory in general, and patients are able to go about their daily lives without having to use a facial epithesis and attracting unwanted attention.

- Psychosocial results

**Figure 3:**
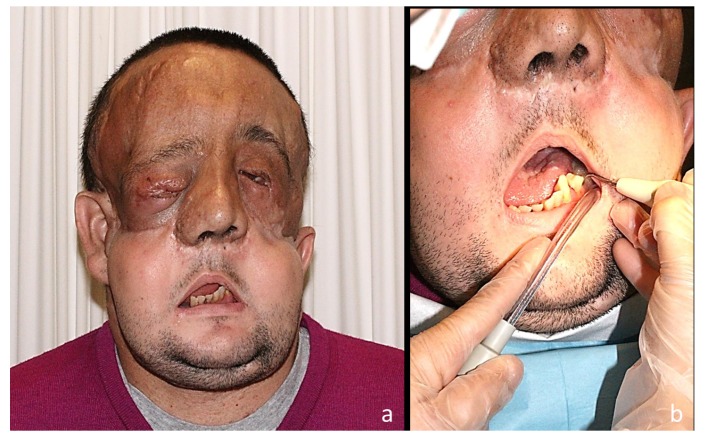
FT for restoration of a severe defect of the lower two thirds of the face after removal of a bilateral massive plexiform neurofibroma of the trigeminal nerve. (A) Clinical outcomes at 2 years, and (B) detail of dental care.

Psychological outcomes in patients after a FT procedure are unknown due to the novel nature of the procedure, although first transplanted patients preliminary results have reported positive results to date. In general, patients experience an acceptable quality of life with a social reintegration and significant changes for having recovered their body image, without psychological disorders (11,35). Lantieri et al. (28) reported quantitative improvements in the quality of life in their first 4 cases. Some patients have even returned to working life. So far, there have been no problems regarding the transfer of identity and the body image changes among FT recipients. After donor’s face placement on the recipient, image results are a combination of both subjects. The new facial appearance is the consequence of combining the different bony structures of each face (Fig. 3).

- Ethical implications 

From an ethical point of view, a widely literature debated issue is the fact that patients need to be under a lifelong immunosuppressive treatment leading to the consequent increased risk of developing complications (2). This fact is therefore important as long as FT is not a surgical procedure that “saves lives” in the same way as heart, lung, or liver transplantation do. From this point of view, the risks of immunosuppressive therapy may outweigh the benefits of the procedure. However, all transplant teams have reported that functional capabilities reestablishment and facial restoration mean “a new life for patients” and that a significant improvement in their quality of life has been experimented (31). One of the unresolved issues remaining would be allograft total loss (2), as long as the resulting aesthetic catastrophic situation could lead to very few reconstructive options for the patient (37).

- Oral considerations in face transplantation

Patients with FT may also need specific dental care that should be considered in the context of immunosuppressed patients (Fig. 3). Meningoud et al. (15) reported a successful case of dental implant placement and dental prosthesis manufacture, arguing that oral rehabilitation was necessary in transplant patients to achieve a functional and aesthetic improvement as well as a normal social reintegration.

Another important aspect is dental occlusion management in allografts that include maxilla and mandible with teeth. Gordon et al. (40) emphasized the importance of ensuring not only the best profile and harmony in the sagittal alignment of the skeleton but to obtain a correct maxillo-mandibular relationship. In order to obtain this result they advocate an orthognathic planning in the FT bone components to achieve a good functional occlusion and a Class I relationship, given the discrepancies that may arise between the maxilla and mandible of the donor and the recipient. Some authors have reported some degree of malocclusion, as one of the patients operated in Paris who underwent a second surgery after FT because dental occlusion was not correct (6). Subsequently, a bilateral jaw osteotomy was then performed.

## Conclusion

FT procedures offer the opportunity to achieve functional, social and aesthetic rehabilitation for patients with severe facial injuries in which conventional reconstructive surgical procedures have failed. Between November 2005 and March 2012 data from 18 clinical cases of FT have been published, but obviously many more transplants will be performed in the near future. Clinical experience has clearly demonstrated the viability of FT as a reconstructive option. However, it remains an experimental procedure, which requires a multidisciplinary approach from basic research and clinical teams. The literature worldwide spread of the first cases results has promoted the evolution from the first partial FT to the full FT procedures, expanding the range of eligible patients. However, unresolved issues and crucial aspects to be defined regarding functionality, quality of life, mid-term and long-term FT complications still remain. All FT teams must be committed to being transparent in communicating surgical experiences. Therefore, FT may become a first-line reconstructive option for patients with severe facial disfigurements. Until now, it has been indicated in a small group of highly selected patients and has been performed only in four countries. In the near future it is expected that the new knowledge transfer from basic research into clinical practice will extend the range of indications and patient access, likely involving more hospitals worldwide in this procedure.
